# Sequence variation and haplotypes of lipoxygenase gene LOX-1 in the Australian barley varieties

**DOI:** 10.1186/1471-2156-15-36

**Published:** 2014-03-19

**Authors:** Hongxia Ye, Stefan Harasymow, Xiao-Qi Zhang, Blakely Paynter, Dianxing Wu, Michael Jones, Xiaoli Shu, Chengdao Li

**Affiliations:** 1 State Key Lab of Rice Biology and Key Lab of the Ministry of Agriculture for Nuclear-Agricultural Sciences, Zhejiang University, Hangzhou 310029, P.R. Chin; 2 Department of Agriculture and Food, Government of Western Australia, 3 Baron-Hay Court, South Perth, WA 6151, Australia; 3 The State Agricultural Biotechnology Centre, Murdoch University, Murdoch, WA 6150, Australia

**Keywords:** Lipoxygenase-1, Structural gene, Haplotype, Malting quality, *Hordeum vulgare*

## Abstract

**Background:**

Lipoxygenases are a family of enzymes which catalyse the hydroperoxidation of polyunsaturated fatty acids with a cis, cis-1,4-pentadiene to form conjugated hydroperoxydienes. Lipoxygenase-1 (LOX-1) in barley worsens the flavour and foam stability of beer. It has become a major selection criteria for malting quality in the last few years.

**Results:**

Lipoxygenase activity was investigated in 41 Australian barley cultivars and advanced breeding lines released since the 1950s; the cultivars differed markedly, ranging from 22.3 to 46.5 U/g. The structural gene and its promoter of lipoxygenase-1 were sequenced from the barley varieties representing different levels of LOX. Based on the analysis of nucleotide and deduced amino acid sequences, two major haplotypes were identified. Barley varieties with lower LOX were classified into three categories based on their pedigrees and sequence variations in the structural gene: (1) barley varieties derived from Canadian varieties with the pre-harvest sprouting susceptible allele, (2) Skiff and Hindmarsh with unique haplotype in the structural gene, and (3) Gairdner and Onslow with an unknown mechanism.

**Conclusion:**

Lipoxygenase activity has been reduced in the malting barley cultivars in the last 60 years although it is only recognized as a malting quality trait recently. There are clear haplotypes of the lipoxygenase structual gene. The polymorphisms detected in the structural gene can be used to design molecular markers for selection of low LOX haplotype. Other mechanisms also existed for controlling lipoxygenase activity. The results suggest that it is possible to develop barley varieties with lower LOX by combination of low LOX-1 haplotype and other trans-regulation factors.

## Background

Lipoxygenases (EC1.13.11.12) are a family of enzymes which catalyse the hydroperoxidation of polyunsaturated fatty acids with a cis, cis-1,4-pentadiene structure (e.g. linoleic, linolenic and arachidonic acids) to form conjugated hydroperoxydienes. Multiple lipoxygenase isoforms exist in plants, which are characterised by different temporal and spatial distributions during plant development [1]. Three isoforms have been described in barley, two of which—LOX-1 and LOX-2—have been purified and characterised from the embryo of germinated seeds. These two isoenzymes clearly differ: LOX-1 presents in both quiescent and germinating barley grains, while LOX-2 appears during germination [2]. LOX-1 produces mainly 9-hydroperoxide from linoleic acid whereas LOX-2 forms primarily 13-hydroperoxide from the same fatty acid. LOX-1 has a relatively low pI compared with LOX-2. The two lipoxygenase cDNA sequences from barley have been characterised and chromosomal locations of the corresponding genes have been determined. LOX-1 is encoded by LoxA mapped on chromosome 4H, while LOX-2 is encoded by LoxC on chromosome 5H [3]. Expression of a third LOX isoform encoded by LoxB, and similar to that of LOX-2, has been detected only after germination and the causal gene has not been identified [4].

LOX-1 provides the predominant LOX activity in the mature seed and malt. It can oxidise malt-derived linoleic acid during mashing to 9-hydroperoxyocatadecadienoic acid (9-HPOD) in the production of malt alcoholic beverages, which is an upstream metabolite of the biochemical pathway leading to formation of trans-2-nonenal (T2N) and trihydroxyoctadecenoic acids (THOD). T2N is the source of a cardboard-like odour (a peculiar foul odour given off by degraded beer drinks) and THODs can cause astringency and decrease foam stability of beer resulting in lower quality malt alcoholic beverages. Consequently, it appears that inhibiting LOX-1 activity can inhibit 9-HPOD production, making it possible to prevent T2N and THOD generation and effectively maintain beer drink quality. Nowadays, methods for reducing LOX activity (especially LOX-1) both during breeding and malting or mashing have been extensively investigated. In fact, brewing scientists and brewers have successfully developed process-controlling methods to improve flavour stability of beer by preventing enzymatic lipid oxidation. However, beer staling might be better controlled by selecting malting barley cultivars with low levels of LOX, engineering coding gene(s) in barley to control expression of the enzymes, or breeding new cultivars with null-LOX activity. Beers made with the lipoxygenase-1-less (LOX-less) malting barley variety had reduced levels of beer-deteriorating substances, such as T2N and THOD compared to beers made with the control malt. The sensory evaluation results indicate that LOX-less barley variety CDC PolarStar improved flavour stability without affecting other beer characteristics [5]. Carlsberg’s research working with Heineken has shown that brewing beer using null-LOX barley minimises negative beer-staling components, provides stable, quality foam with no aged off-flavours, and keeps its fresh flavour for longer (http://www.null-lox.co.uk/).

Baxter [6] first suggested that barley LOX was a genotypic characteristic. Yang and Schwarz [7] suggested that kilning condition and genotypic variation may impact LOX isoenzyme survival in finished malt. Wu *et al*. [8] found significant genotypic variation in LOX-1 activity in several barley cultivars, suggesting that LOX-1 activity may be reduced through breeding.

To date, several null/low LOX-1 barley lines have been identified. Hirota *et al*. [9] discovered six LOX-1 null barley lines by surveying many landrace lines. These lines did not show any significant LOX-1 activity and lacked the authentic LOX-1 protein. Genetic analysis revealed that this null-LOX-1 trait was governed by a single recessive gene located at the LoxA locus on chromosome 4H. The six LOX-1 null mutants shared similar features and the same unique polymorphism in a structural gene region, implying that these mutants might be derived from the same ancestral origin. Through mutation breeding, Oozeki *et al*. [10] obtained a LOX-1-deficient mutant line ‘Daikei LM1’ from sodium azide treated ‘Karl’ M2. DNA sequence analysis of the LOX-1 gene of ‘DaikeiLM1’ revealed that a single nucleotide substitution (C to T) resulted in a stop codon in the third exon of LOX-1. Using LOX-1 null barley in brewing can effectively improve flavour stability in beer without changing other important beer qualities [11]. A LOX-less commercial barley variety CDC PolarStar was successfully developed by successive backcross and molecular marker assisted selection of the mutant gene [12]. In addition, studies have shown that the Lox1 5′-untranslated leader sequence was involved in embryo-specific expression; its leader sequence contains cis-elements regulating qualitative (tissue-specific) and quantitative gene expression and an enhancer element that increases activity of upstream promoter fragments in vivo [13]. These studies indicate that the structural gene of LOX-1 plays a key role in determining LOX content.

LOX content is also important in other crops. For example, LOX play a role in the development of unpleasant flavors in foods containing soybean by oxidation of polyunsaturated fatty acids. Null-LOX mutants have also been developed for soybean breeding [14].

In this study, we surveyed LOX activity among Australian barley varieties by sequencing the promoter and structural gene of Lox1 which represents different levels of LOX activity. We analysed the Lox1 gene nucleotide and deduced amino acid sequences, compared mutation sites among null-LOX-1 mutants, and determined its phylogenetic relationship among these varieties. Our investigation aimed to further understand genetic control of LOX activity, and provide a theoretical foundation for developing low-LOX malting barley cultivars, which may in turn improve the flavour stability of some beers.

## Methods

### Materials

The genetic material included 36 of the 65 barley varieties released in Australia since the 1950s and 5 advanced breeding lines (Additional file 1). All the materials are two-rowed spring type. A sub-set of varieties—*Hindmarsh*, *Gairdner*, *Skiff*, *Onslow*, *Clipper*, *Franklin*, *Moondyne* and *Chevalier*—were selected to investigate the sequence variation of Lox1 gene based on their LOX activity (see Lipoxygenase Assay section below).

### Field trials

The field plot was planted in a randomised complete block design in plots 1 × 3 m^2^. Plants were grown at three sites in Western Australia with two replicates at each site. Harvested grains from each site were evaluated for their physical quality, and grains from the best site were used for micro-malting analysis.

### Micro-malting analysis

Barley samples were cleaned and sieved over a 2.2 mm screen prior to micro-malting in a Joe White Systems micro-malting unit without additives. A standard malting schedule was used by steeping at 19°C for 7 h wet, 8 h air rest, 3 h wet, 4 h air rest, 1 h wet. Germination took 96 h (48 h at 18°C followed by 48 h at 16°C), then moisture adjusted to 46% for 24 h. Kilning was 2 h at 45°C, 3 h at 50°C, 4 h at 55°C, 3 h at 60°C, 3 h at 65°C, 3 h at 70°C, 2 h at 75°C, and 4 h at 80°C. Malt rootlets were removed using a custom-made rootlet-removing machine (Fraser Fabrications P/L, Malaga, Western Australia).

### Lipoxygenase assay

We used the Joe White Malting revised version of the Malt Lipoxygenase (LOX) (original assay from Baxter [6]). All processes were completed on ice unless otherwise indicated.

### Preparation of substrate solution (2.5 linoleic acid)

Five milliliters of 0.05 M borate buffer (pH 9.0) was added to a volumetric flask (10 mL) followed by 0.25 mL Tween20, 0.25 mL linoleic acid and 0.65 mL 1 M NaOH. The contents were shaken gently in an ultrasonic bath with ice water until the solution became clear, then distilled water was added to 10 mL.

### Enzyme extraction from finished malt

Finished malts were milled in a Retsch ZM200 centrifugal mill (Retsch GmBH, Germany) with a 1.0 mm screen; 5 g milled malt was transferred to 100 mL flask. 50 mL of acetate buffer (pH 5.0) containing 0.1 M NaCl was added and kept in ice water bath for 15 min with occasional shaking. The resulting solution was transferred to a 1.5 mL eppendorf tube and centrifuged for 5 min at 10 000 rpm. The supernatant was subsequently transferred to a new eppendorf tube and stored on ice.

### Enzyme assay

The temperature of the cell holder and phosphate buffer (0.1 M, pH 6.8) was equilibrated to 25°C by water circulation. 100 μL enzyme extract and 2850 μL phosphate buffer (0.1 M, pH 6.8) was added to 50 μL substrate solution, mixed, returned to the cell holder and absorbance recorded at 1 min and 4 min at 234 nm. Blank absorbance was measured using 50 μL substrate solution and 2950 μL phosphate buffer at 1 min.

### Calculation and expression of results

One unit of LOX activity represents an increase in absorbance at 234 nm per minute, per gram malt on dry basis, as calculated using the following formula:

$$\begin{array}{c} \mathrm{LOX}\;\mathrm{activity}=\frac{\mathrm{Abs}\;\left(4\min\right)–\mathrm{Abs}\;\left(1\min\right)}{3}\\ \;\times \frac{1}{5\times \left(100–M/100\right)}\times \frac{50\;000}{B} \end{array}$$

where Abs (4 min) is absorbance at 234 nm at 4 min of reaction, Abs (1 min) is absorbance at 234 nm at 1 min reaction, M is moisture content of malt sample, and B is volume (μL) of enzyme extract of malt used.

### DNA extraction

Genomic DNA was extracted from the leaves of two-week-old seedlings for each variety following the phenol chloroform standard protocol. DNA samples were quantified using the Nanodrop and adjusted to a final concentration of about 25 ng/μL for PCR analysis.

### Primer design and PCR amplification for isolation of Lox1 promoter and gene

The full-length Lox1 promoter sequence has been isolated from Himalaya [15] (NCBI database, U83904). Full-length cDNAs of the Lox1 gene have been isolated from barley varieties Triumph [3] (NCBI database, L35931) and Haruna Nijo (NCBI database, AK252639). Based on the sequences from the NCBI database (http://www.ncbi.nlm.nih.gov), PCR primers were designed for sequencing with 100–200 bp overlaps between adjacent fragments (Additional file 2).

PCR reactions were carried out in a final volume of 50 μL containing about 100 ng of genomic DNA, 10× PCR buffer, 1.5 mM MgCl_2_, 200 μM of each dNTP, 400 nmol of each primer, and one unit of Taq DNA polymerase. The reaction was initially denatured at 95°C for 5 min, followed by 35 cycles of 95°C for 30 s, annealing for 45 s and 72°C for 1 min. The PCR was terminated at 72°C for 7 min.

### Transformation, cloning and sequencing

The PCR-amplified DNA was excised separately from agarose gels and extracted using QIAEX II Gel Extraction (QIAGEN). The DNA sequence was ligated into pGEM®-T Easy Vectors (Promega) according to manufacturer recommendations. Competent *E. coli* cells, strain JM109, were transformed and the clones screened by blue/white colony selection. Plasmid DNA from the colony was isolated by the alkali lysis method [16]. Recombinant DNA was screened for appropriate insert size by digestion with EcoRI restriction enzyme. The pGEM-T Easy cloned products were sequenced with primers M13F and M13R. Sequencing reactions were performed on PCR products according to manufacturer recommendations, using Big-Dye labeling sequencing reaction mixture. Sequences were read on an Applied Biosystems 3730 DNA sequencer (SABC, Murdoch University, Western Australia).

### Data analysis

Sequences were edited to remove the vector sequence and extra restriction sites. The promoter region and intron–exon structure of the barley LOX-1 coding region were deduced by comparing the nucleotide sequence of the barley published Lox1 cDNA sequence (NCBI database L35931 and AK252639). The amino acid sequence of Lox1 was deduced using Translate Tools in the ExPaSy web server (http://au.expasy.org/tools/dna.html). Multiple alignments of sequences were used with Clustal X (v 1.82) and performed using GeneDoc (v2.5). Phylogenetic reconstructions were done using the MEGA4.0 package. Distance matrices were constructed using the Kimura two-parameter model and trees constructed using the neighbour-joining algorithm. The dataset was re-sampled 1000 times using the bootstrap method. Phylogenetic analyses are presented for the Lox1 DNA sequences.

## Results

### Phenotypic variation of LOX in australian barley varieties

**Figure 1:**
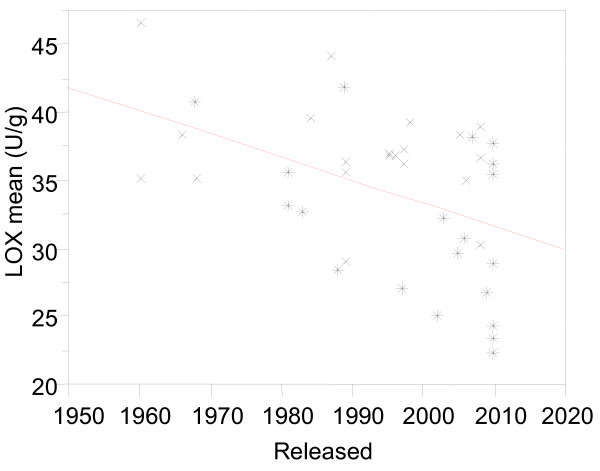
**Variation trends of LOX content in 41 Australian barley cultivars over the last 60 years (*R*** = -0.46**,*P* < **0.01).** The ‘x’ indicated feed cultivars and the ‘*’ indicated malting cultivars.

Forty-one barley cultivars and advanced breeding lines were surveyed for LOX activity, which included major barley varieties grown in Australia since 1950s (Additional file 1). LOX content varied markedly among the 41 Australian barley cultivars and advanced breeding lines, ranging from 22.3 to 46.5 U/g. LOX content has gradually decreased in Australian barley cultivars over the last 60 years (Figure 1; *R* = -0.46**, *P* < 0.01), although there is markedly variation. On average, malting cultivars had about 17 less LOX compared with feed varieties (*P* < 0.01). Hindmarsh, Gairdner, Onslow, Skiff, Hamelin, Flagship, WABAR2480, WABAR2481 and WABAR2482 had the lowest LOX and could be used to develop barley varieties with lower levels of LOX.

### Nucleotide sequence of Lox1 promoter

The previous study demonstrated that cis-acting elements within the untranslated leader sequence of the Lox1 gene are essential for embryo-specific expression, and that it is possible to modify tissue-specificity of other genes by inserting this leader sequence. Deletion/replacement analysis of the Lox1 leader sequence, combined with transient expression in germinating embryos and *in vitro* transcription/translation assays, suggests that essential promoter or enhancer element(s) involved in Lox1 expression in embryos are located between -129 and -75 bp [13]. In this study, 1101 bp (from -1076 to +12) Lox1 promoter fragment sequences were obtained from eight different barley varieties with different LOX contents. The sequences of these eight varieties were compared with the Himalaya promoter sequence (NCBI database, U83904) [15]. Alignment showed that the Australian varieties differ from the Himalaya variety. The eight sequenced varieties shared high degrees of identity. Franklin had one base pair difference at position -807 (Himalaya -795) (C → T) and Skiff had one variation at position -702 (Himalaya -690) (A → G). These variations are located outside the known functional domain (data not shown) [13].

### Analysis of Lox1 structural gene

**Figure 2:**
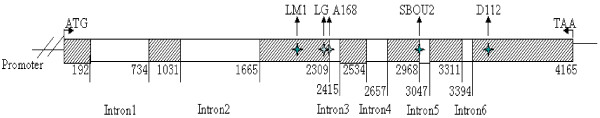
**Gene structure of Lox1 gene — composed of 7 exons (stippled boxes) and 6 introns (white boxes)—and the mutation positions in five null/low LOX-1 mutant lines.** Stars indicate mutation positions.

Lox1 sequence contains 7 exons and 6 introns, and the length of the complete Lox1 sequence is 4165 bp (Moondyne, Vintage Onslow, Gairdner, Franklin, Chevalier, Barke, Neruda) (Figure 2) or 4188 bp (Clipper, Hindmarsh, Skiff. The 4188 bp variant, not shown in Figure 2, has 9 bp and 16 bp insertion in intron1 and intron 2, respectively, and 2 bp deletion in intron 3 compared with the 4165 bp variant.

**Figure 3:**
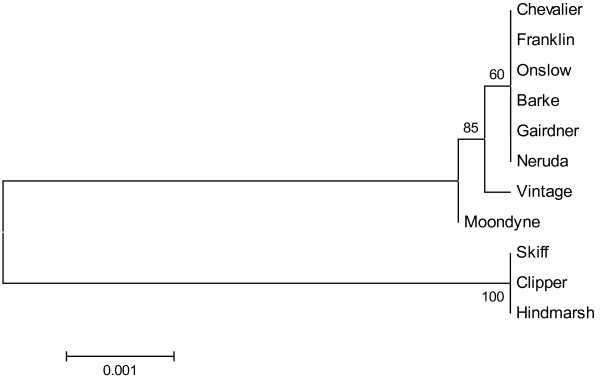
Phylogenetic relationships of Lox1 gene.

Lox1 gene sequences of 11 varieties were used for alignment in this study, including eight varieties representing different LOX levels (high/low). The others were the wild types (Vintage (AX469865), Neruda (CS172656), Barke (CS172652)) of the low/null LOX-1 mutants (Line G (AX469868), A168 (CS172657), D112 (CS172653)), (http://wheat.pw.usda.gov/GG2/index.shtml). Forty-three variant positions were identified among these 11 sequences (Additional file 3). Alignment results identified two types of sequences: (1) those with the same sequence, which included Skiff, Clipper and Hindmarsh, and (2) those which shared a similar sequence, except Vintage and Moondyne with a few variations. A phylogenetic analysis of the 11 varieties is presented in Figure 3. The phylogenetic tree indicates that the Lox1 gene sequence of Skiff, Clipper and Hindmarsh had the same origin; Lox1 gene of Chevalier, Franklin, Onslow, Barker, Gairdner, Neruda, Vintage and Moondyne may belong to a different origin.

### Analysis of coding regions

**Figure 4:**
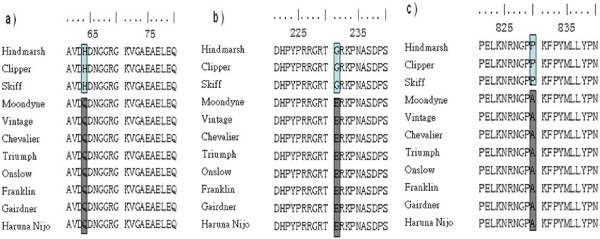
Alignment results among amino acid sequences deduced from different plasmid sequencing in this paper and others published in Graingene2.

The cDNA sequences of Triumph (L35931), Haruna Nijo (AK252639) and Vintage (AX469847) were retrieved from the NCBI database (http://www.ncbi.nlm.nih.gov). cDNA sequences of the eight barley varieties were deduced from the sequenced genomic DNA. Alignment of the cDNA sequences among these varieties identified 19 single nucleotide substitutions (Additional file 3). Three single nucleotide replacements resulted in amino acid substitutions His/Gln-64, Gly/Glu-231, Pro/Ala-830 in the Lox1 gene coding region (Figure 4). The alignment of the deduced amino acid sequences further supports the two distinguished haplotypes of Lox1 gene in the current barley varieties. Further study is required to understand relationship of the amino acid substitutions and LOX activity. Comparing nucleotide sequences with the wild type LOX-1 gene, all null-LOX-1 lines—Daikei LM1, A168, D112 and SBOU2—shared a single nucleotide substitution [9], [10], which introduced an internal stop codon (Figure 2, Additional file 4) (Patent WO02053721). The low LOX-1 barley, Line G, had two nucleotide substitutions in 2 exons, one of which (at position +2279) led to a non-conservative 368 amino acid substitution (Glycine → Aspartate) in the expressed protein (Patent WO02053721). The insertion of a charged amino acid residue at this position is thus likely to disturb the structural and functional properties of the enzyme.

## Discussion

In the brewing industry, LOX-1 is regarded as a negative enzyme that worsens the flavour and foam stabilities of beer. Therefore, a barley cultivar with lower LOX-1 content or heat-labile LOX-1 is preferable for brewing. A previous study suggested that LOX activity is significantly (*P* > 0.05) affected by cultivar, while interactions of environment × cultivar are not significant [8]. Both the level of gene expression and the stability and specific activity determine the level of enzyme activity in the tissue.

Hirota *et al*. [17] identified a major quantitative trait locus (QTL) for LOX activity on chromosome 4H, which was located at the same locus as the structural gene (Lox1), explaining 82 of phenotypic variance. A minor QTL was detected on chromosome 7H. More recently, Jin *et al*. [18] found another 3 QTLs controlling LOX content in the Stirling/Harrington DH population. One QTL at chromosome 5HL contributed to 39 of genetic variation in LOX content. The second QTL, close to the centromere region of chromosome 5H, accounted for 17 of genetic variation. A minor QTL on chromosome 2H explained 6 of genetic variation but was significant in both years. These studies indicate that the structural gene of LOX-1 plays a key role in determining LOX content. However, LOX content can also be controlled by other genes or trans-regulation.

In the present study, we surveyed LOX content in Australian commercial barley varieties from the 1950s. In general, malting barley varieties have less LOX than feed varieties, and modern varieties had less LOX than older varieties (Figure 1; Additional file 1). As LOX content has not been a selection criterion in breeding programs to date, we speculate that low LOX is associated with other preferred malting quality traits. This phenomenon has also been observed in QTL mapping for many traits including enzyme activity for alpha-amylase and beta-glucanase [18]. A recently study demonstrated 9-lipoxygenase as modulator of local and systemic defense in Arabidopsis. The lox1 mutant enhanced susceptibility and partially impaired activation of systemic acquired resistance [19]. Further study is required to understand if the selection of low LOX enhanced disease susceptibility in the good malting quality barley variety, e.g. Baudin.

Nine barley cultivars and breeding lines—Hindmarsh, Skiff, Gairdner, Onslow, Hamelin, Flagship, WABAR2480, WABAR2481 and WABAR2482—were identified with lower LOX contents. Hamelin, Flagship, WABAR2480, WABAR2481 and WABAR2482 shared common ancestry with variety Harrington. It is likely that these varieties and breeding lines have common genes for controlling low LOX. In a previous study, two QTLs were identified from Harrington controlling low LOX content on chromosome 5HC and 5HL [18]. As there is no LOX structural genes in the chromosomal regions, the low LOX content from Harrington and its derived varieties should be due to trans-regulation. The same regions were also reported to regulate the activity of alpha-amylase and beta-glucanase [20]. Furthermore, the two QTL coincided with the QTLs for seed dormancy/pre-harvest sprouting. As the pre-harvest sprouting susceptible alleles were associated with low LOX content, these varieties may only be used for breeding low-LOX varieties in areas with low risk of pre-harvest sprouting [18].

Onslow is one of the parents of Gairdner; we speculate that low LOX in these two varieties share a common mechanism. These two varieties are reasonably tolerant to pre-harvest sprouting, so may have a different mechanism for low LOX than the other varieties. Sequence analysis showed high identity of the LOX-1 gene in these two varieties as well as the normal-LOX varieties (Figure 4; Additional file 3). Further research is needed to understand the low-LOX mechanism in Gairdner and Onslow.

Hindmarsh and Skiff have a unique LOX-1 gene sequence compared with other varieties. These included one 10 bp insertion, one 17 bp insertion, one 2 bp deletion and 39 single nucleotide substitutions (Additional file 3) which resulted in three amino acid changes (Figure 4). It is not clear if these variations are associated with LOX content, as the third variety Clipper had the same sequence, but different LOX content. Other mechanisms may be involved in controlling LOX content in Clipper.

Thermostability is another key factor to determine final LOX content in malt. Segregation analysis revealed that LOX thermostability types in the Steptoe/Morex DHLs were governed by a single locus, located at the same locus as the LOX-1 structural gene of chromosome 4H. It is clear that the factor controlling LOX thermostability types was located at the LoxA locus which corresponds with the LOX-1 structural gene [4]. To understand the diversity in the thermostability of seed lipoxygenase-1 (LOX-1), Hirota *et al*. [21] investigated 1040 cultivars of barley (*Hordeum vulgare* ssp. *vulgare*): the relative thermostability of LOX-1 (LOX-RTS) in these lines had a bimodal frequency distribution and were categorised into high and low thermostability types (H-type and L-type, respectively). Thus, it is likely that thermostability is controlled by the structural variation of LOX-1 as demonstrated in beta-amylase [22] Testing is underway to see if the three amino acid substitutions in Hindmarsh, Skiff and Clipper result in changes in thermostability (Figure 4).

## Conclusion

Lipoxygenase activity has been reduced in the malting barley varieties in the last 60 years although it is only recognized as a malting quality trait recently. In general, malting barley varieties have less LOX than feed varieties, and modern varieties had less LOX than older varieties. There are clear haplotypes of the lipoxygenase structual gene, which is an important factor for controlling lipoxygenase activity. The polymorphisms detected in the structural gene can be used to design molecular markers for selection of low LOX haplotype. Further study is required to understand the relationship between the haplotypes and LOX activity or thermostability. But other mechanisms also existed for controlling lipoxygenase activity. The results suggest that it is possible to develop barley varieties with lower LOX by combining different mechanisms.

## Abbreviations

Aa: Amino acid; HPOD: Hydroperoxide; LOX: Lipoxygenase; NJ: Neighbor-joining algorithm; Nt: Nucleotide; THOD: Trihydroxyoctadecenoic acid.

## Competing interests

The author’s declare that they have no competing interests.

## Authors’ contributions

HY: data analysis and draft the paper; SH: micromalting and enzyme assays; XZ: DNA sequencing; BP: conducted field trial; DW: experimental design; MJ: supervised experiment and draft paper; XS: data analysis; CL: data analysis and finalized the paper.

## Supplementary Material

Additional file 1 List of barley cultivars used in this study including their origins and type. Click here for file

Additional file 2 PCR primers for amplification of DNA fragments. Click here for file

Additional file 3 List of variable positions in the Lox1 sequence of different barley varieties. Click here for file

Additional file 4 Development of low/null-LOX malting barley cultivars. Click here for file
