# Supplementary material for: Sequence variation and haplotypes of lipoxygenase gene LOX-1 in the Australian barley varieties
Source: BMC Genet. 2014 Mar 19;15:36. doi: 10.1186/1471-2156-15-36 (PMC4003807; doi:10.1186/1471-2156-15-36)
Supplement: Additional file 1 — List of barley cultivars used in this study including their origins and type. [file 1471-2156-15-36-S1.docx]

Additional file 1 List of barley cultivars used in this study including their origins and type

| Cultivar | Pedigree | LOX activity  (U/g) | | Type |  |
| --- | --- | --- | --- | --- | --- |
| Barque | Triumph/Galleon | | 36.2 | Feed | |
| Baudin | Stirling/Franklin | | 32.3 | Malt | |
| Buloke | Franklin/VB9104//VB9104 | | 38.3 | Malt | |
| Bussell | Prior/Ymer | | 35 | Feed | |
| Capstan | Waveney/WI2875//Chariot/Chebec | | 38.3 | Feed | |
| Chevalier | English Land Race | | 46.5 | Feed | |
| Clipper | Prior-A/Proctor | | 40.9 | Malt | |
| Commander | WI2976/Sloop//Galaxy | | 28.9 | Malt | |
| Dampier | Olli/Research | | 38.3 | Feed | |
| Dash | Chad/Joline//Cask | | 36.7 | Feed | |
| Doolup | XBVT210/3/Prior/Lenta//Noyep/Lenta/Dampier | | 39.2 | Feed | |
| Fitzgerald | Onslow/Tas 85-466 | | 37.1 | Feed | |
| Flagship | Chieftan/Barque//Manley/VB9104 | | 29.7 | Malt | |
| Fleet | Mundah/Keel//Barque | | 30.2 | Feed | |
| Forrest | Newal/Peatland//OAC-21 | | 35.1 | Feed | |
| Franklin | Shannon/Triumph | | 42 | Malt | |
| Gairdner | Onslow/Tas 85-466 | | 27.1 | Malt | |
| Hamelin | Stirling/Harrington | | 34.9 | Malt | |
| Hannan | WABAR2023//Windich/Morex | | 25.2 | Feed | |
| Harrington | Klages/3/Gazelle/Betzes//Centennial | | 33.2 | Malt | |
| Hindmarsh | Dash/VB9409 | | 26.8 | Malt | |
| Lockyer | Tantangara/VB9104 | | 36.6 | Feed | |
| Molloy | GoldenPromise/WI2395/4/(72S:267)XBVT210/3/Atlas57//Prior/Ymer/O’Connor | | 36.8 | Feed | |
| Moondyne | Dampier/Prior//Ymer/Parwan | | 44.1 | Feed | |
| Mundah | O'Connor/Yagan | | 36.9 | Feed | |
| O'Connor | Proctor/CI3576(WI2231)/3/(XBVT212)Atlas57//(A14)Prior/Ymer | | 39.5 | Feed | |
| Onslow | Forrest/Aapo | | 28.9 | Feed | |
| Prior | Chevalier Selection | | 34.1 | Feed | |
| Roe | Doolup//Windich/Morex | | 38.9 | Feed | |
| Schooner | Proctor/Prior-A//Proctor/CI-3576 | | 32.7 | Malt | |
| Skiff | Abed-Deba/3/Proctor/CI-3576//CPI-18197/Beka/4/Clipper/Diamant//Proctor/CI-3576 | | 28.5 | Malt | |
| Stirling | Dampier//(A-14)Prior/Ymer/3/Piroline | | 35.7 | Malt | |
| VB0432 | Scarlett/Gairdner | | 37.8 | Malt | |
| Vlamingh | WABAR0570/TR118 | | 30.7 | Malt | |
| WABAR 2480 | WB229/4*Hamelin | | 22.3 | Malt | |
| WABAR 2481 | WB229/4*Hamelin | | 23.5 | Malt | |
| WABAR 2482 | WB229/4*Hamelin | | 24.5 | Malt | |
| WABAR2312 | WABAR2023/Alexis | | 35.5 | Malt | |
| WABAR2315 | WABAR2023/Alexis | | 39.4 | Malt | |
| Windich | ATLAS-57/Prior//Ymer/Parwan | | 36.2 | Feed | |
| Yagan | unknown | | 35.5 | Feed | |
