# Supplementary material for: Sequence variation and haplotypes of lipoxygenase gene LOX-1 in the Australian barley varieties
Source: BMC Genet. 2014 Mar 19;15:36. doi: 10.1186/1471-2156-15-36 (PMC4003807; doi:10.1186/1471-2156-15-36)
Supplement: Additional file 2 — PCR primers for amplification of DNA fragments. [file 1471-2156-15-36-S2.doc]

Additional file 2 PCR primers for amplification of DNA fragments

| Primer name | Primer sequence | Amplified size (bp) | Primer postion | Annealing temperature |
| --- | --- | --- | --- | --- |
| LoxAP | 5’ATTAGGTTGAATGAGACTTTGCC3’  5’ TCCCAGCAGCATCTTGTTCT3’ | 1088 | -1076～+12 | 58 |
| LoxA1 | 5’ GCAGTGAAAGCGAGGAGA 3’  5’ TCGTGGAGGGTGATGGTT 3’ | 968(977) | -34～+934(+943) | 58 |
| LoxA2 | 5’ GACGGGGGAGTCCAAGTT3’  5’ GTGGTGTCCACGTAGGTG3’ | 1238(1253) | +808(817) ～+2046(2070) | 60 |
| LoxA3 | 5’ AAGGCTGGGTGTGGGA 3’  5’ GCCGTCGTTCGGGTAG 3’ | 650 | +2891(2914)～+3541(3564) | 65 |
| LoxA4 | 5’ CCATCACGCAGGGCATCCTG 3’  5’ GCGTTGATGAGCGTCTGCCG 3’ | 1197(1195) | +1996(2021)～+3193(3216) | 65 |
| LoxA5 | 5’ TACAAGGTGCGGTTGCTG3’  5’ TTGCCGATGGCTTAGATT 3’ | 757 | +3424(3447) ～+4181(4204) | 65 |
