# Supplementary material for: Sequence variation and haplotypes of lipoxygenase gene LOX-1 in the Australian barley varieties
Source: BMC Genet. 2014 Mar 19;15:36. doi: 10.1186/1471-2156-15-36 (PMC4003807; doi:10.1186/1471-2156-15-36)
Supplement: Additional file 3 — List of variable positions in the Lox1 sequence of different barley varieties. [file 1471-2156-15-36-S3.doc]

Additional file 3 List of variable positions in the LoxA sequence of different barley varieties

| Varieties | Sites of variation | | | | | | | | | | | | | | | | | | | | | |
| --- | --- | --- | --- | --- | --- | --- | --- | --- | --- | --- | --- | --- | --- | --- | --- | --- | --- | --- | --- | --- | --- | --- |
| DNA | 159 | 192 | 335 | 558 | 646 | 668 | 691 | 815 | 863 | 986 | 1004 | 1040 | 1117 | 1133 | 1174 | 1136 | 1472 | 1487 | 1521 | 1531 | 1540 | 1549 |
| cDNA | 159 | 192 |  |  |  |  |  | 273 | 321 | 444 | 462 |  |  |  |  |  |  |  |  |  |  |  |
| Hindmarsh | A | C | T | G | GAACTC  GACT | G | G | A | G | T | C | T | C | C | C | A | G | C | G | G | G | TACATT  CTAAA  ATATGT |
| Skiff | A | C | T | G | GAACTC  GACT | G | G | A | G | T | C | T | C | C | C | A | G | C | G | G | G | TACATT  CTAAA  ATATGT |
| Clipper | A | C | T | G | GAACTC  GACT | G | G | A | G | T | C | T | C | C | C | A | G | C | G | G | G | TACATT |
|  |  |  |  |  |  |  |  |  |  |  |  |  |  |  |  |  |  |  |  |  |  | CTAAA  ATATGT |
| Moondyne | C | A | C | A | G | G | T | G | C | C | A | A | A | G | G | G | A | C | A | A | C | T |
| Vintage | C | A | C | A | G | G | T | G | C | C | A | A | A | G | G | G | A | T | A | A | C | T |
| Onslow | C | A | C | A | G | A | T | G | C | C | A | A | A | G | G | G | A | C | A | A | C | T |
| Gairdner | C | A | C | A | G | A | T | G | C | C | A | A | A | G | G | G | A | C | A | A | C | T |
| Franklin | C | A | C | A | G | A | T | G | C | C | A | A | A | G | G | G | A | C | A | A | C | T |
| Chevalier | C | A | C | A | G | A | T | G | C | C | A | A | A | G | G | G | A | C | A | A | C | T |
| Barke | C | A | C | A | G | A | T | G | C | C | A | A | A | G | G | G | A | C | A | A | C | T |
| Neruda | C | A | C | A | G | A | T | G | C | C | A | A | A | G | G | G | A | C | A | A | C | T |

| Varieties | Sites of variation | | | | | | | | | | | | | | | | | | | | |
| --- | --- | --- | --- | --- | --- | --- | --- | --- | --- | --- | --- | --- | --- | --- | --- | --- | --- | --- | --- | --- | --- |
| DNA | 1552 | 1561 | 1563 | 1596 | 1704 | 1868 | 1896 | 2040 | 2103 | 2319 | 2341 | 2347 | 3005 | 3233 | 3237 | 3260 | 3293 | 3791 | 4064 | 4066 | 4072 |
| cDNA |  |  |  |  | 528 | 692 | 720 | 864 | 927 |  |  |  |  | 1740 | 1744 | 1767 | 1800 | 2215 | 2488 | 2490 | 2496 |
| Hindmarsh | A | T | C | A | A | G | T | T | A | A | - | A | C | T | C | T | C | C | C | C | C |
| Skiff | A | T | C | A | A | G | T | T | A | A | - | A | C | T | C | T | C | C | C | C | C |
| Clipper | A | T | C | A | A | G | T | T | A | A | - | A | C | T | C | T | C | C | C | C | C |
| Moondyne | C | C | T | G | G | A | C | C | G | G | GT | G | T | C | T | C | G | G | G | C | T |
| Vintage | C | C | T | G | G | A | C | C | G | G | GT | G | T | C | T | C | G | G | G | T | T |
| Onslow | C | C | T | G | G | A | C | C | G | G | GT | G | T | C | T | C | G | G | G | T | T |
| Gairdner | C | C | T | G | G | A | C | C | G | G | GT | G | T | C | T | C | G | G | G | T | T |
| Franklin | C | C | T | G | G | A | C | C | G | G | GT | G | T | C | T | C | G | G | G | T | T |
| Chevalier | C | C | T | G | G | A | C | C | G | G | GT | G | T | C | T | C | G | G | G | T | T |
| Barke | C | C | T | G | G | A | C | C | G | G | GT | G | T | C | T | C | G | G | G | T | T |
| Neruda | C | C | T | G | G | A | C | C | G | G | GT | G | T | C | T | C | G | G | G | T | T |
