# Supplementary material for: Sequence variation and haplotypes of lipoxygenase gene LOX-1 in the Australian barley varieties
Source: BMC Genet. 2014 Mar 19;15:36. doi: 10.1186/1471-2156-15-36 (PMC4003807; doi:10.1186/1471-2156-15-36)
Supplement: Additional file 4 — Development of low/null-LOX malting barley cultivars. [file 1471-2156-15-36-S4.docx]

Additional file 4 Development of low/null-LOX malting barley cultivars

| Mutant | Wild Type/  Control | Gene | | | Protein length in amino acids | Reference |
| --- | --- | --- | --- | --- | --- | --- |
|  |  | Change | Position in genomic DNA | Result |  |  |
| Daikei LM1 | Karl (WT) | C→T | 2005 | Translation stop | － | (Oozeki et al. 2007) |
| Line G | Vintage (WT) | G→A | 2279 | Translation error | 862 | Patent WO02053721 |
| A168 | Neruda (WT) | G→A | 2311 | RNA splicing error; Translation stop | 399 | Patent WO2005087934 |
| D112 | Barke (WT) | G→A | 3574 | Translation stop | 655 | Patent WO2005087934 |
| SBOU2 | Vintage (Control) | G→A | 2968 | RNA splicing error; Translation stop | － | Patent US20080193593 (Hirota et al. 2005) |
